# Associations of anthropometric measures on breast cancer risk in pre- and postmenopausal women—a case-control study

**DOI:** 10.1186/s40101-016-0090-x

**Published:** 2016-03-08

**Authors:** Renata Pacholczak, Wiesława Klimek-Piotrowska, Piotr Kuszmiersz

**Affiliations:** Department of Anatomy, Jagiellonian University Medical College, ul. Kopernika 12, 31-034 Cracow, Poland

**Keywords:** Breast cancer, Anthropometry, Obesity, BMI, WHR

## Abstract

**Background:**

The type of silhouette and quantity of fat tissue are correlated with hormonal imbalance which plays a substantial role in breast carcinogenesis. The goal of the study was to investigate the association between various anthropometric characteristics and breast cancer risk.

**Methods:**

Detailed anthropometric assessment was conducted on 487 women of whom 193 had diagnosed breast cancer and were consecutive patients in the Oncology Center, Cracow, Poland between 2002 and 2004. Measurements were divided into four categories: overall body size (body mass index [BMI], waist circumference [WC], waist-hip ratio [WHR]), regional body sizes (skinfold thicknesses, circumferences), thickness of the skeleton (widths, chest diameters), and body proportions. Additionally, results were analyzed in regard to menopausal status. Differences between groups were assessed using Student’s *t* test and Mann-Whitney’s test. Models of logistic regression for selected data were built to estimate the odds ratio. Results were considered statistically significant when the *P* value was less than 0.05.

**Results:**

The BMI in both groups was negatively associated with the risk of cancer. Among premenopausal women, WHR increased the risk of breast cancer (WHR > 0.83, OR, 2.72; 95 % CI, 1.01–7.27). Anthropometric indices of hip-to-shoulder ratio in postmenopausal (≥84.2 mm, OR, 0.02; 95 % CI, 0.01–0.11) and trunk-to-height ratio in both premenopausal women (≥32.76, OR, 0.09; 95 % CI, 0.03–0.28) and postmenopausal women (≥32.76, OR, 0.13; 95 % CI, 0.05–0.33) were strongly related to a decreased risk of breast cancer. Thicknesses of the triceps and subscapular skinfolds increased the risk of breast cancer.

**Conclusions:**

Women with breast cancer present with an obese type of silhouette with a specific concentration of fat tissue in the central and upper parts of the body.

## Background

There are many studies which confirm that breast cancer is strongly associated with body size [1–7]. Characteristics which are generally taken into account when assessing body shape include height, weight, body mass index (BMI), waist-hip ratio (WHR), and waist circumference (WC) [1–5, 8, 9]. All these measurements are found to be correlated with menopausal status, and their value is linked to estrogen and androgen levels [10–14]. However, results of different studies remain discordant.

Notably, peripheral fat tissue plays a substantial role in the synthesis of sex steroid hormones in women. Thus, after menopausal transition, obesity leads to a higher level of estrogen and androgens which are mitogenic agents for breast cells. Equally, it has been found that the moment of diagnosis of breast cancer in obese women is delayed and chemotherapy is less efficient [15]. Moreover, obesity is a sign of bad dietary habits and lack of physical activity which are considered as risk factors in breast carcinogenesis [16].

The question remains whether there are any different features in the body silhouette which may exemplify hormonal imbalance in women with breast cancer. In this aim, we conducted detailed anthropometric studies of 488 women of whom 193 had diagnosed breast cancer. The parameters which we considered may be divided into four categories: overall body size (BMI, WC, WHR), regional body sizes (skinfold thickness, circumferences), thickness of the skeleton (widths, chest diameters), and body proportions.

We compared our results with different studies; however, thickness of the skeleton and body proportions have not hitherto been described in the literature. We also hypothesized that body composition differences in breast cancer women are only a symptom of different metabolic and biochemical disturbances. Therefore, certain anthropometric measurements could be helpful in identifying women at a higher risk of developing breast cancer in the future.

## Material and methods

We performed a case-control population study based on 488 women—193 cases and 295 control subjects. The manuscript submitted for publication contains an experiment which complies with the current law of Poland where it was performed and was approved by the Ethics Committee Board in Cracow, Poland.

### Participants

All cases were collected from the Oncology Center, Cracow, Poland. We included patients aged between 35 and 80 years old with newly diagnosed breast cancer and who were qualified for radical mastectomy during the period of 2002–2004. Each patient’s diagnosis was confirmed by pathologists. We excluded patients with a previous history of cancer, after neoadjuvant treatment, pregnant, breastfeeding, or with liver disease. Female controls were chosen randomly from local industry companies, public education institutes, and social service workers. Cancer-free participants were matched to patients by age and sex to eliminate the most important confounding factors.

### Data collection

Data were collected anonymously and consent from all participants was obtained orally. After taking all measurements, each patient signed up on a questionnaire as an approval of participation in the study.

All participants were interviewed by students and the authors in person at the hospital and workplace and via a structured questionnaire to obtain such information as demographic factors, menstrual status, reproductive history with a detailed history of breast feeding, and family history of cancer. Menopause was defined as no menses for 12 months or bilateral oophorectomy or hysterectomy.

Anthropometric measurements were directly taken in accordance with International Standards for Anthropometric Assessment [17] by trained interviewers with (1) digital scales to measure weight (without clothes and shoes); (2) a large anthropometer (which measures between 0 and 60 cm in 0.1-cm increments) to localize specific anthropometric marks (such as basion, vertex, symphysion, lumbal, acromial, iliocristal, and suprasternal marks); (3) Martin breadth calipers to measure hip width (biiliocristal diameter between the outer edges of the iliac crest); shoulder width (biacromial diameter); wrist, elbow, knee, and ankle widths (all measurements were taken at midpoints); and arm width (at a mid-acromiale-radiale point); (4) chest depth caliper to measure transverse and sagittal chest diameter (at nipple level); (5) anthropometric non-stretchable tape to measure height (without shoes, looking straight ahead, with shoulders down, with participants standing next to the wall), waist circumference (at the midpoint between the lower margin of the last palpable rib and the top of the iliac crest), hip circumference (at the widest part of the buttocks on the level of greater trochanter), neck circumference (on the level of the laryngeal prominence), and arm, thigh, and wrist circumferences at midpoints; and (6) skinfold caliper to measure triceps skinfold thickness (at the midpoint of the back of the upper arm), subscapular skinfold thickness (at the lower angle of the scapula), suprailiac skinfold thickness (at the one quarter of the distance between the navel and anterior superior iliac spine), and calf skinfold thickness (at midpoint). In order to take the mentioned measurements, we used Mikropolis’ anthropometric measuring kit.

To estimate body proportion, we calculated body mass index (BMI by dividing weight [kg] with the square of height [m]), waist-to-hip ratio (WHR), and additional anthropometric indexes such as the length of the trunk (from suprasternal mark to symphysion), hip-to-shoulder ratio, hip-to-height ratio, shoulder-to-height ratio, and trunk-to-height ratio.

### Data analysis

All statistical analyses were stratified according to menopausal status. To evaluate differences between cases and control participants, Student’s *t* test and Mann-Whitney’s test were conducted for demographic factors, reproductive history and all taken anthropometric measurements. We performed the analysis for normal distribution for every single measurement, and then, we used a *t* test for parameters presenting normal distribution (namely thigh circumference, arm width, wrist width) and Mann-Whitney U test for these parameters which did not qualify for the *t* test (the rest of the parameters). Furthermore, logistic regression analysis was performed for the following anthropometric factors: hip-to-shoulder ratio; trunk-to-height ratio; suprailiac, triceps, and subscapular skinfold thicknesses; and wrist circumference and for BMI and WHR ratios to obtain the odds ratios (OR) with 95 % confidence intervals. To observe the dose-response effect, we categorized all these variables into quartile groups. Additionally, we adjusted each analysis for age, education status, and potential confounders: positive family history of breast cancer, age at menarche, number of parity, and age of menopause for the postmenopausal group. All analyses were performed using STATISTICA 10.0. Results were considered statistically significant when the *P* value was less than 0.05.

## Results

Analysis of the baseline characteristic of the groups (Table 1) showed statistically significant differences between all participants in age at enrollment, age at last birth, number of parity, family history of breast cancer in first-degree relatives, and number of miscarriages. Patients with breast cancer were significantly older than cancer-free women (56.2 ± 11.8 vs 50.4 ± 13.2 years, *P* < 0.01) and were older at the time of the birth of their last offspring (30.1 ± 6.1 vs 28.2 ± 4.9 years, *P* < 0.01). They had a greater number of parity (2.0 ± 1.4 vs 1.5 ± 1.1, *P* < 0.01) and more episodes of miscarriages (0.7 ± 2.2 vs 0.4 ± 0.7, *P* < 0.01). A positive family history of breast cancer in first-degree relatives was reported more often by healthy individuals (83 vs 26, *P* < 0.01). We did not observe any statistical differences regarding age at menarche, age at first birth, education, and age at menopause.

**Table 1:** Comparison of cases and controls on demographics and selected breast cancer risk factors

	Premenopausal women			Postmenopausal women			All women		
	*n* = 239			*n* = 248					
	Cases (61) mean value (SD)	Controls (178) mean value (SD)	*P* value	Cases (132) mean value (SD)	Controls (116) mean value (SD)	*P* value	Cases (193) mean value (SD)	Controls (294) mean value (SD)	*P* value
Age at enrollment (years)	45.1 (5.3)	41.8 (6.1)	<0.01	61.4 (10.4)	63.4 (10.2)	0.2	56.2 (11.8)	50.4 (13.2)	<0.01
Age at menarche (years)	14.0 (1.6)	13.9 (1.4)	0.7	14.1 (1.6)	14.1 (1.9)	0.7	14.1 (1.6)	14.0 (1.6)	0.8
Age at first birth (years)	24.4 (4.5)	24.2 (4.1)	1.0	24.7 (4.3)	24.6 (4.5)	0.7	24.6 (4.4)	24.3 (4.2)	0.6
Age at last birth (years)	29.3 (7.1)	27.7 (1.9)	0.7	30.5 (5.5)	29.5 (5.1)	0.3	30.1 (6.1)	28.2 (4.9)	<0.01
Number of parity	1.9 (1.3)	1.5 (0.9)	0.7	2.1 (1.4)	1.5 (1.3)	<0.01	2.0 (1.4)	1.5 (1.1)	<0.01
Family history of breast cancer in first-degree relatives	10	49	0.07	16	34	<0.01	26	83	<0.01
Education			0.29			0.13			<0.01
Primary	15	33		57	34		72	67	
Vocational	7	31		5	8		12	39	
Secondary	21	47		49	48		70	94	
Higher	18	67		21	25		39	92	
Number of miscarriages	1.0 (3.7)	0.3 (0.7)	0.1	0.6 (1.0)	0.5 (0.8)	0.4	0.7 (2.2)	0.4 (0.7)	<0.01
Age at menopause (years)	–	–	–	48.7 (5.2)	49.5 (4.7)	0.8	48.7 (5.2)	49.5 (4.7)	0.8

In the group of premenopausal participants, significant differences were observed only in age at enrollment because of the greater number of participants in the control group (cases were older than controls 45.1 ± 5.3 vs 41.8 ± 6.1 years, *P* < 0.01). In postmenopausal participants, there were no differences in age and the only statistically significant differences were connected with the number of parity (2.1 ± 1.4 cases vs 1.5 ± 1.3 controls, *P* < 0.01) and with a positive family history of breast cancer (16 cases vs 34 controls, *P* < 0.01).

An analysis of anthropometric measurements (Table 2) showed a number of differences in the body structure between women with breast cancer and healthy participants. A comparison without stratification according to menopausal status revealed that cancer participants had greater chest—transverse and sagittal—diameters; thigh, wrist, and waist circumferences; wrist, elbow, ankle, and arm widths; and triceps, subscapular, and calf skinfold thicknesses measurements. Measurements of pelvis diameters and length of trunk were significantly lower. Estimation of body proportions revealed that women with breast cancer have narrower pelvises, broader shoulders, and shorter trunks compared to their height.

**Table 2:** Comparison of anthropometric measurements of cases and controls

	Premenopausal women			Postmenopausal women			All women		
	*n* = 239			*n* = 248			*n* = 487		
	Cases (61) mean value (SD)	Controls (178) mean value (SD)	*P* value	Cases (132) mean value (SD)	Controls (116) mean value (SD)	*P* value	Cases mean value (SD)	Controls mean value (SD)	*P* value
Overall body size									
Body mass (kg)	67.98 (13.06)	65.45 (12.16)	0.28	69.47 (12.24)	70.18 (12.11)	0.71	69.0 (12.49)	67.32 (12.34)	0.14
Height (m)	1.59 (0.06)	1.59 (0.06)	0.38	1.56 (0.06)	1.55 (0.06)	0.20	1.57 (0.06)	1.58 (0.06)	0.16
WHR	0.80 (0.05)	0.76 (0.05)	<0.01	0.81 (0.05)	0.81 (0.07)	0.21	0.81 (0.05)	0.78 (0.07)	<0.01
BMI	27.14 (5.96)	25.59 (4.78)	0.26	28.48 (4.54)	29.14 (4.46)	0.99	28.07 (5.06)	26.99 (4.96)	0.05
Waist circumference (cm)	82.21 (10.68)	78.92 (10.53)	0.05	87.06 (10.61)	89.25 (12.0)	0.30	85.52 (10.84)	82.99 (12.21)	<0.01
Hip circumference (cm)	103.23 (9.46)	103.53 (9.07)	0.85	106.86 (10.75)	109.5 (10.13)	0.08	105.71 (10.47)	105.89 (9.93)	0.95
Regional body sizes									
Arm circumference (cm)	29.19 (4.24)	28.87 (3.34)	0.87	30.05 (3.64)	31.12 (3.74)	0.03	29.78 (3.85)	29.76 (3.67)	0.66
Thigh circumference (cm)	56.13 (5.2)	54.58 (5.31)	0.05	55.70 (5.12)	53.59 (6.56)	0.01	55.83 (5.13)	54.19 (5.83)	<0.01
Wrist circumference (cm)	16.26 (1.06)	15.91 (0.92)	0.02	16.95 (1.08)	16.55 (1.14)	0.01	16.72 (1.12)	16.16 (1.06)	<0.01
Neck circumference (cm)	34.58 (2.87)	35.61 (2.21)	0.21	35.02 (2.47)	35.45 (2.35)	0.29	34.88 (2.6)	35.54 (18.0)	0.10
Triceps skinfold thickness (mm)	30.50 (9.39)	24.52 (7.4)	<0.01	30.03 (8.96)	27.84 (7.46)	0.21	30.19 (9.07)	25.84 (7.59)	<0.01
Subscapular skinfold thickness (mm)	32.32 (12.68)	29.42 (11.44)	0.12	35.35 (11.06)	33.72 (12.51)	0.08	34.4 (11.65)	31.12 (12.04)	<0.01
Suprailiac skinfold thickness (mm)	34.35 (12.81)	34.06 (14.05)	0.88	38.57 (11.7)	42.73 (15.44)	0.09	37.23 (12.19)	37.49 (15.2)	0.80
Calf skinfold thickness (mm)	20.44 (5.74)	17.87 (4.62)	0.02	19.88 (5.22)	18.97 (5.24)	0.33	20.04 (5.35)	18.3 (4.89)	0.01
Thickness of the skeleton									
Hip width (biiliocristal) (mm)	59.35 (3.81)	58.05 (4.6)	0.04	61.04 (5.64)	62.73 (5.23)	0.02	60.52 (5.19)	59.9 (5.36)	0.08
Shoulder width (biacromial) (mm)	76.53 (4.65)	73.86 (4.84)	<0.01	76.50 (6.38)	73.35 (5.33)	<0.01	76.51 (5.88)	73.66 (5.04)	<0.01
Wrist width (mm)	52.54 (2.97)	51.66 (3.1)	0.17	54.07 (3.18)	53.77 (3.4)	0.58	53.6 (3.18)	52.48 (3.38)	0.01
Elbow width (mm)	71.80 (7.46)	64.38 (5.1)	<0.01	72.50 (9.93)	66.84 (5.76)	<0.01	72.31 (9.28)	65.36 (5.5)	<0.01
Knee width (mm)	93.81 (9.53)	92.78 (6.73)	0.32	97.03 (8.89)	96.79 (8.1)	0.83	96.03 (9.16)	94.32 (7.53)	0.07
Ankle width (mm)	66.13 (3.47)	63.90 (3.67)	<0.01	66.59 (4.08)	64.96 (3.84)	0.01	66.45 (3.88)	64.31 (3.77)	<0.01
Arm width (mm)	76.29 (3.55)	74.51 (3.86)	<0.01	77.60 (3.76)	74.88 (3.9)	<0.01	77.19 (3.74)	74.65 (3.87)	<0.01
Transverse chest diameter (mm)	278.25 (21.52)	267.13 (20.92)	<0.01	276.61 (21.16)	275.27 (23.11)	0.63	277.13 (21.22)	270.34 (22.13)	<0.01
Sagittal chest diameter (mm)	202.92 (23.28)	198.76 (21.96)	0.33	218.16 (23.14)	219.87 (23.04)	0.76	213.37 (24.18)	207.09 (24.62)	<0.01
Maximal pelvis diameter (mm)	342.20 (25.4)	346.52 (28.98)	0.30	345.36 (23.39)	362.64 (27.87)	<0.01	344.37 (24.02)	352.88 (29.57)	<0.01
Body proportions									
Length of trunk (mm)	487.02 (26.72)	503.22 (36.71)	<0.01	489.60 (70.27)	500.81 (36.56)	<0.01	488.79 (60.14)	502.27 (36.61)	<0.01
Hip-to-shoulder ratio	77.55 (4.74)	78.74 (6.04)	0.24	79.97 (5.86)	85.69 (6.46)	<0.01	79.21 (5.63)	81.48 (7.07)	<0.01
Hip-to-height ratio	18.20 (1.55)	18.29 (1.42)	0.44	18.99 (1.35)	20.20 (1.51)	<0.01	18.74 (1.46)	19.04 (1.73)	0.05
Shoulder-to-height ratio	23.44 (1.33)	23.25 (1.06)	0.20	23.78 (1.16)	23.6 (1.08)	0.26	23.67 (1.23)	23.39 (1.08)	<0.01
Trunk-to-height ratio	30.69 (1.96)	31.57 (2.09)	<0.01	31.39 (4.72)	32.29 (2.06)	<0.01	31.17 (4.07)	31.86 (2.11)	<0.01

Among premenopausal woman with breast cancer, we observed significantly higher measurements in shoulders, elbow, ankle, arm, transverse chest diameter, wrist and thigh circumferences, triceps skinfold thicknesses, calf skinfold thicknesses, and WHR. While in the group of postmenopausal women with breast cancer, we found significant differences in shoulder, arm, elbow, pelvis, and ankle breadths and thigh and wrist circumferences and they presented with broader arms and shorter trunks.

It seems that hip width, suprailiac skinfold thickness, and arm and neck circumferences are not correlated either with menopausal status or breast cancer.

Among premenopausal women, WHR was associated with an increased risk of breast cancer (between subjects in the highest quartile WHR > 0.83, adjusted OR, 2.72; 95 % CI, 1.01–7.27). However, in postmenopausal women, there was no linear association between WHR value and cancer risk. BMI in both groups was negatively associated with cancer risk; nevertheless, OR values were not statistically significant (Table 3).

**Table 3:** Association between anthropometric measurements and breast cancer risk

	Premenopausal						Postmenopausal						All women					
	Cases		Controls		OR (95 % CI)	*P* value	Cases		Controls		OR (95 % CI)	*P* value	Cases		Controls		OR (95 % CI)	*P* value
	No.	Percent	No.	Percent			No.	Percent	No.	Percent			No.	Percent	No.	Percent		
BMI																		
<23.8	20	33.9	64	36.0	1.0 (reference)		23	17.5	14	12.1	1.0 (reference)		43	22.6	78	26.5	1.0 (reference)	
≥23.9–<27.4	17	28.8	58	32.6	0.68 (0.31–1.50)	0.3	30	22.9	25	21.5	0.64 (0.25–1.62)	0.3	47	24.7	83	28.2	0.73 (0.42–1.27)	0.3
≥27.4–<30.3	10	17.0	31	17.4	0.61 (0.23–1.58)	0.1	34	26.0	39	22.6	0.49 (0.20–1.19)	0.3	44	23.2	70	23.8	0.61 (0.34–1.11)	0.1
≥30.3	12	20.3	25	14.0	0.70 (0.26–1.90)	0.1	44	33.6	38	32.8	0.49 (0.20–1.19)	0.5	56	29.5	63	21.5	0.72 (0.40–1.31)	0.3
WHR																		
<0.75	10	17.8	74	41.8	1.0 (reference)		7	5.8	21	18.1	1.0 (reference)		17	9.6	95	32.4	1.0 (reference)	
≥0.75–<0.79	15	26.8	56	31.6	1.04 (0.43–2.53)	0.9	28	23.1	20	17.2	1.65 (0.66–4.12)	0.3	43	24.3	76	25.9	1.51 (0.84–2.70)	0.2
≥0.79–<0.83	16	28.6	28	15.8	2.01 (0.78–5.18)	0.1	42	34.7	29	25.0	1.81 (0.76–4.35)	0.2	58	32.8	57	19.5	2.25 (1.23–4.13)	0.01
≥0.83	15	26.8	19	10.8	2.72 (1.01–7.27)	0.04	44	36.4	46	39.7	0.97 (0.42–2.24)	0.9	59	33.3	65	22.2	1.50 (0.81–2.76)	0.2
Hip-to-shoulder ratio																		
<75.9	21	35.0	64	36.0	1.0 (reference)		34	26.0	2	1.7	1.0 (reference)		55	28.8	66	22.4	1.0 (reference)	
≥75.9–<80.3	24	40.0	50	28.1	1.19 (0.56–2.54)	0.6	32	24.4	17	14.7	0.10 (0.02–0.50)	0.01	56	29.3	67	22.8	0.69 (0.39–1.22)	0.2
≥80.3–<84.2	11	18.3	37	20.8	0.61 (0.24–1.52)	0.3	38	29.0	35	30.2	0.06 (0.01–0.30)	0.01	49	25.7	72	24.5	0.42 (0.23–0.75)	0.01
≥84.2	4	6.7	27	15.2	0.35 (0.10–1.25)	0.1	27	20.6	62	53.4	0.02 (0.01–0.11)	0.01	31	16.2	89	30.3	0.14 (0.08–0.30)	0.01
Trunk-to-height ratio																		
<30.17	25	42.4	41	23.0	1.0 (reference)		39	29.8	15	12..9	1.0 (reference)		64	33.7	56	19.0	1.0 (reference)	
≥30.17–<31.28	11	18.6	45	25.3	0.28 (0.11–0.70)	0.01	37	28.2	28	24.2	0.45 (0.20–1.04)	0.06	48	25.3	73	24.8	0.45 (0.26–0.79)	0.01
≥31.28–<32.76	15	25.4	43	24.2	0.25 (0.10–0.64)	0.01	36	27.5	29	25.0	0.35 (0.15–0.82)	0.02	51	26.8	72	24.5	0.41 (0.24–0.74)	0.01
≥32.76	8	13.6	49	27.5	0.09 (0.03–0.28)	0.01	19	14.5	44	37.9	0.13 (0.05–0.33)	0.01	27	14.2	93	31.6	0.14 (0.08–0.27)	0.01
Suprailiac skinfold thickness																		
<27.5	16	26.7	63	35.6	1.0 (reference)		23	17.7	14	12.0	1.0 (reference)		39	20.5	77	26.3	1.0 (reference)	
≥27.5–<36.5	19	31.7	37	20.9	1.79 (0.77–4.16)	0.2	33	25.4	29	25.0	0.57 (0.23–1.39)	0.2	52	27.4	66	22.5	1.22 (0.69–2.16)	0.5
≥36.5–<45.5	17	28.3	38	21.5	1.16 (0.48–2.84)	0.7	36	27.7	35	30.2	0.53 (0.22–1.25)	0.1	53	27.9	73	24.9	0.94 (0.53–1.66)	0.8
≥45.5	8	13.3	39	22.0	0.44 (0.44–1.25)	0.1	38	29.2	38	32.8	0.49 (0.21–1.16)	0.1	46	24.2	77	26.3	0.78 (0.44–1.39)	0.4
Triceps skinfold thickness																		
<22	14	23.3	67	37.9	1.0 (reference)		13	10.0	25	21.6	1.0 (reference)		27	14.2	92	31.4	1.0 (reference)	
≥22–<27	8	13.3	42	23.7	0.79 (0.29–2.15)	0.6	29	22.3	29	25.0	1.47 (0.61–3.56)	0.4	37	19.5	71	24.2	1.31 (0.71–2.42)	0.4
≥27–<33	19	31.7	42	23.7	1.89 (0.82–4.36)	0.1	38	29.2	28	24.1	1.98 (0.84–4.70)	0.1	57	30.0	70	23.9	1.92 (1.08–3.42)	0.03
≥33	19	31.7	26	14.7	2.64 (1.08–6.47)	0.03	50	38.5	34	29.3	2.24 (0.96–5.21)	0.06	69	36.3	60	20.5	2.56 (1.43–4.56)	0.01
Subscapular skin-fold thickness																		
<23.7	17	28.3	60	33.9	1.0 (reference)		20	15.3	24	20.7	1.0 (reference)		37	19.4	84	28.7	1.0 (reference)	
≥23.7–<31.5	11	18.3	54	30.5	0.62 (0.25–1.53)	0.3	22	16.8	32	27.6	0.69 (0.29–1.65)	0.4	33	17.3	86	29.3	0.64 (0.35–1.15)	0.1
≥31.5–<40	16	26.7	28	15.8	1.30 (0.52–3.24)	0.6	49	37.4	29	25.0	1.77 (0.78–4.03)	0.2	65	34.0	57	19.5	1.72 (0.98–3.02)	0.06
≥40	16	26.7	35	19.8	1.06 (0.43–2.57)	0.9	40	30.5	31	26.7	1.25 (0.55–2.85)	0.2	56	29.3	66	22.5	1.28 (0.73–2.26)	0.4
Wrist circumference																		
<15.5	9	16.4	50	28.4	1.0 (reference)		6	5.3	21	18.3	1.0 (reference)		15	8.9	71	28.4	1.0 (reference)	
≥15.5–<16.25	17	30.9	60	24.1	0.72 (0.30–1.73)	0.5	26	23.7	23	20.0	0.83 (0.35–1.99)	0.7	44	26.0	83	28.5	0.84 (0.47–1.49)	0.5
≥16.25–<17	14	25.4	38	21.6	0.68 (0.26–1.78)	0.4	22	19.3	24	20.9	0.60 (0.25–1.44)	0.3	36	21.3	62	21.3	0.81 (0.44–1.50)	0.5
≥17	15	27.3	28	15.9	0.90 (0.32–2.49)	0.8	59	51.7	47	40.8	0.88 (0.41–1.89)	0.7	74	43.8	75	25.8	1.16 (0.66–2.04)	0.6

We also observed a significant impact of the hip-to-shoulder ratio on cancer risk in postmenopausal women. High values of this factor were strongly associated with a decreased risk of breast cancer (in the last quartile ≥84.2, adjusted OR, 0.02; 95 % CI, 0.01–0.11). Furthermore, increased trunk-to-height ratio was also significantly associated with a decreased risk of breast cancer among both premenopausal women (in the last quartile ≥32.76, adjusted OR, 0.09; 95 % CI, 0.03–0.28) and postmenopausal women (in the last quartile ≥32.76, adjusted OR, 0.13; 95 % CI, 0.05–0.33). Thicknesses of triceps and subscapular skinfolds were associated with an increased risk of breast cancer, and wrist circumference was associated with a decreased risk; however, these factors did not reach statistical significance (Table 3).

## Discussion

The link between anthropometric measurements and breast cancer risk in different studies remains uncertain [1–7]. In addition, results are influenced by multiple factors such as ethnicity, lifestyle, and reproductive status [6]. Overall, it is well established that obesity has an impact on breast cancer occurrence. It is important to distinguish two types of obesity—android (WHR > 0.8) in which fat is mainly distributed in the upper body (shoulders, abdomen) and gynoid (WHR < 0.8) where fat accumulates in the lower part of the body (buttocks, thighs) [18]. In persons with android obesity, metabolic and hormonal abnormalities are more pronounced.

In our data, pre- and postmenopausal women are overweight (with BMI > 25 kg/m^2^) without statistically significant differences between groups. According to our investigations, central obesity increases the risk of developing breast cancer in premenopausal women—these data are consistent with previous studies [1–3, 7, 17]. However, conversely to previous reports [2–5, 7], in postmenopausal women, its effect is neutral. It may be due to a general tendency toward android obesity in elderly women caused by decreasing levels of estrogen. Despite these factors, bad dietary habits and a lack of physical activity after menopausal transition also lead to visceral obesity.

In postmenopausal women, peripheral adipocytes are the source of all estrogens which are synthesized from the conversion of androstenedione. It means that general obesity is directly linked to an increased level of mitogenic factors within cancer cells [8, 10, 19]. Obese premenopausal women have lower estrogen levels due to the storage of estrogen in fatty tissue, decrease in ovarian activity, and frequent anovulatory cycles [1, 2]. Thus, general obesity is considered as a protection for breast cancer in premenopausal women. In contrast to previous investigations [3–7, 20], our study did not reveal a correlation between BMI and the risk of breast cancer or any statistical differences in BMI among both pre- and postmenopausal women.

The harmful effect of central obesity remains complex. This type of obesity is correlated with insulin resistance, increased level of insulin-like growth factor (IGF)-1, and fatty acids [2, 3, 7, 8, 21]. Insulin inhibits hepatic production of sex hormone-binding globulin (SHBG), elevates leptin levels, and diminishes adiponectin levels (both with angiogenic effect) [22]. Furthermore, insulin modulates VEGF expression [3, 15]. Together, these substances promote mammary carcinogenesis by accelerating cell divisions and inducing the synthesis of transcriptional factors. Obesity also induces “smoldering” inflammation causing a permanently elevated level of cytokines (TNF alpha, IL-6, CRP) which promote tumurigenesis [18, 22, 23].

In regard to body shape, women with breast cancer present an android type of silhouette with the distribution of fat tissue in the central and upper parts of the body which is revealed by increased shoulder width, waist circumference, skinfold thickness of the triceps and scapula, and differences in body proportions. Anthropometric indicators showed that women with breast cancer have narrower hips and shorter trunks and are shorter with broader arms—features which are typical for men. In premenopausal women, such characteristics are not so evident; however, they deteriorate after menopause. This is due to the high level of androgens postmenopause caused by a stimulation of androgen synthesis by IGF-1 in ovarian theca cells and a lower level of SHBG. It has been stated that the level of free testosterone increases with BMI [8, 10, 13] especially in android obesity [18, 24]. Androgens may promote growth by directly binding to the androgen receptors or estrogen receptors in breast tissue or via conversion to estrogen in either peripheral or breast fat tissue [14].

Statistically significant differences in the thickness of the skeleton between premenopausal and postmenopausal women with breast cancer are an unusual finding which has previously not been described in the literature. Compared to healthy individuals, breast cancer women had elevated widths of the wrist, elbow, knee, arm, and ankle and a bigger thorax. The most substantial event during menopausal transition is decreasing ovarian activity resulting in deficient estrogen levels. The latter causes many menopausal symptoms such as a loss of bone mass [25]. It is highly probable that in overweight women this situation is inverted. Constant higher estrogen exposure during lifetime contributes to faster bone maturation which begins in adolescence. There is also an impact of the elevated level of IGF-1 (one of the mitogenic factors) [26]. In adults, estrogen diminishes bone resorption by inhibition of osteoclasts. In overweight women with breast cancer, this effect is amplified by elevated levels of androgens which also have beneficial effects on bone tissue [27]. Androgens act either by conversion to estrogen via p450 aromatase enzyme complex or by directly binding to androgen receptors. Interestingly, androgens are responsible for the periosteal expansion of bones which may be a direct explanation of our findings [27]. However, it is necessary to mention that central obesity leading to metabolic complications and inflammation may be also associated with poor bone health [28].

### Study limitations and future research

The anthropometric measures used in the present study have some limitations. Firstly, BMI is not a homogenous parameter especially in comparison to modern techniques used in order to assess fat distribution such as the following: dual-energy X-ray absorptiometry (DXA), bioelectrical impedance analysis (BIA), or air displacement plethysmography. It may be misleading in individuals with normal BMI but a high content of body fat. Secondly, WHR may be also an inadequate index as it is under the influence of confounding factors such as age-related variation in muscle mass and tone.

Anthropometric measurements may be miscalculated due to bias during examination (problems in taking some measurements, non-cooperative patients, etc.). The present results are confounded by variables such as differences in age between groups, different stage of the disease, and the method used for selecting the control group. Nonetheless, the present results highlight significant body parameter changes which could be of great value in assessing breast cancer risk.

The present study highlighted some interesting avenues of future research. The bone strengths between overweight breast cancer women are another area which has yet to be explored in depth. Furthermore, the influence of testosterone on breast tissue is an interesting issue particularly after consideration of its estrogen-independent role which merits further investigation.

## Conclusion

Obesity has reached an epidemic level. It should be a matter of great concern due to the negative health complications it is associated with such as higher cancer rates. Our study revealed that women with breast cancer present with a typical android silhouette and visceral obesity, which is more common in men, along with altered body proportions and increased thickness of the skeleton. Some simple anthropometric characteristics (WHR; BMI; skinfold thicknesses; widths of shoulders, elbow, ankle, and wrist; and circumferences of thigh and wrist) and the estimation of body proportions may be a valuable tool for women assessment in order to disclose groups at a higher risk of developing breast cancer in the future.
